# Reviewer acknowledgement

**DOI:** 10.1186/s13041-016-0208-4

**Published:** 2016-03-08

**Authors:** Bong-Kiun Kaang, Min Zhuo, Tim Bliss

**Affiliations:** Seoul National University, Seoul, Republic of Korea; University of Toronto, Toronto, Canada; The Francis Crick Institute, London, UK

## Abstract

The editors of *Molecular Brain* would like to thank all our reviewers who have contributed to the journal in Volume 8 (2015).

**Marisela Agudelo**

United States of America

**Yoshiki Arakawa**

Japan

**Valeria Avdoshina**

United States of America

**Nagi Ayad**

United States of America

**Kasum Azim**

United States of America

**Tudor C Badea**

United States of America

**Daehyun Baek**

Republic of Korea

**Angel Barco**

Spain

**Arnab Barik**

United States of America

**Mark Bear**

United States of America

**Oren Becher**

United States of America

**Guoqiang Bi**

China

**Haruhiko Bito**

Japan

**Vadim Bolshakov**

United States of America

**Victor Borrell**

Spain

**Denis Burdakov**

United Kingdom

**Timothy Bussey**

United Kingdom

**Marco Canossa**

United States of America

**Mian Cao**

China

**L Cao**

United States of America

**Shuwen Cao**

United States of America

**Francesco Cardona**

Italy

**Martine Cattarelli**

France

**Sebastiano Cavallaro**

Italy

**Sunghoe Chang**

Republic of Korea

**Brian Chen**

Canada

**Gong Chen**

United States of America

**Xuanmao Chen**

United States of America

**Youjun Chen**

United States of America

**Zheyu Chen**

China

**Kei Cho**

United Kingdom

**Dh Cho**

Republic of Korea

**Youngshik Choe**

Republic of Korea

**Soo Young Choi**

Republic of Korea

**Kimberly Christian**

United States of America

**Steven Clarke**

United States of America

**Daniel Cohen**

France

**Graham Collingridge**

United Kingdom

**Jaqueline Crawley**

United States of America

**Wim Crusio**

France

**Fernando De Castro**

Spain

**Jessica Deslauriers**

Canada

**Yuqiang Ding**

China

**Tr Doeppner**

Germany

**Bo Duan**

United States of America

**Sebastian Dworkin**

Australia

**Marina Emborg**

United States of America

**Jose Antonio Esteban**

Spain

**Cinthia Farina**

Italy

**André Fischer**

Germany

**Patrick Fisher**

Denmark

**Masayo Fujita**

Japan

**Masaki Fukata**

Japan

**Tomoyuki Furuyashiki**

Japan

**Peter Gass**

Germany

**Anamitra Ghosh**

United States of America

**Peter Giese**

United Kingdom

**Christian Johannes Gloeckner**

Germany

**Kerui Gong**

United States of America

**Johannes Graeff**

Swaziland

**Kamalesh Guliak**

India

**Camilla Gustafsen**

Denmark

**Hideo Hagihara**

Japan

**Jin-Hee Han**

Canada

**Kihoon Han**

Republic of Korea

**Young-Goo Han**

United States of America

**Daniela Hartl**

Germany

**Yasunori Hayashi**

Japan

**Yasunori Hayashi**

United States of America

**Yiping He**

United States of America

**Karl Herrup**

China

**Hajime Hirase**

Japan

**Xiu-Ti Hu**

United States of America

**Andrew Huang**

Taiwan, Republic of China

**Eunmi Hur**

Republic of Korea

**Jeong-Jin Hwang**

Republic of Korea

**Sun Wook Hwang**

Republic of Korea

**Su-Kyeong Hwang**

Republic of Korea

**Kazutaka Ikeda**

Japan

**Masashi Ikeda**

Japan

**Masai Ishii**

Japan

**Anthony Isles**

United Kingdom

**Shigeyoshi Itohara**

Japan

**Takuji Iwasato**

Japan

**Deok-Jin Jang**

Republic of Korea

**Ru-Rong Ji**

United States of America

**Zhengping Jia**

Canada

**Seonmi Jo**

Republic of Korea

**Francois Jouret**

Belgium

**Yong-Keun Jung**

Republic of Korea

**Bong-Kiun Kaang**

Republic of Korea

**Wataru Kakegawa**

Japan

**Roger Kamm**

United States of America

**Fusao Kato**

Japan

**Satoshi Kida**

Japan

**Eun-Kyoung Kim**

Republic of Korea

**Hyong Kyu Kim**

Republic of Korea

**Janghwan Kim**

Republic of Korea

**Jaesang Kim**

Republic of Korea

**Eunjoon Kim**

Republic of Korea

**Sang Jeong Kim**

Republic of Korea

**Byung Gon Kim**

Republic of Korea

**Jin Kim**

Republic of Korea

**Jung-Woong Kim**

Republic of Korea

**Hye-Sun Kim**

Republic of Korea

**Sang Jeong Kim**

Republic of Korea

**Hyoung Kim**

United States of America

**Yang-Hee Kim**

Republic of Korea

**Janine Kirby**

United Kingdom

**Eric Klann**

United States of America

**Shira Knafo**

Spain

**Jaewon Ko**

Republic of Korea

**Kohei Koga**

Canada

**Schichi Koizumi**

Japan

**Ja Wook Koo**

Republic of Korea

**Hisatsugu Koshimizu**

Japan

**Chandra Kothapalli**

United States of America

**Kiyoshi Kurata**

Japan

**Miguel Lafarga**

Spain

**Catherine Lawrence**

United Kingdom

**Kyungmin Lee**

Republic of Korea

**Jae-Hyung Lee**

Republic of Korea

**Jin-A Lee**

Republic of Korea

**Sung Joong Lee**

Republic of Korea

**Yong-Seok Lee**

Republic of Korea

**Jeong Ho Lee**

Republic of Korea

**Seungbok Lee**

Republic of Korea

**Seung-Hee Lee**

Republic of Korea

**Jaekwang Lee**

Republic of Korea

**Jong Eun Lee**

Republic of Korea

**Seungkyu Lee**

United States of America

**Seung-Jae Lee**

Republic of Korea

**Yong-Seok Lee**

Republic of Korea

**Weidong Li**

China

**Minghua Li**

United States of America

**Shihua Li**

United States of America

**He Li**

United States of America

**Xiao-Jiang Li**

United States of America

**F Liao**

United States of America

**Chae Seok Lim**

Republic of Korea

**Fang Liu**

Canada

**Jose Lopez-Atalaya**

Spain

**Daniel Lubelski**

United States of America

**Katarzyna Lukasiuk**

Poland

**Zhenzhong Ma**

United States of America

**D Ma**

United States of America

**Toshiya Manabe**

Japan

**Isabel Martinez**

United States of America

**Fernando Martinez-Garcia**

Spain

**Angeles Martin-Requero**

Spain

**Miho Matsumata**

Japan

**Thomas Mchugh**

Japan

**Britt Mellstrom**

Spain

**Karine Merienne**

France

**Valerie Mezger**

France

**Tsuyoshi Miyakawa**

Japan

**Elek Molnar**

United Kingdom

**Inhee Mook-Jung**

Republic of Korea

**Flavio Moroni**

Italy

**Kirsten Müller-Vahl**

Germany

**Karim Nader**

Canada

**Maria Da Graça Naffah-Mazzacoratti**

Brazil

**Shin Nakagawa**

Japan

**Yasushi Nakagawa**

United States of America

**Kazutoshi Nakazawa**

United States of America

**Jose R. Naranjo**

Spain

**Ferdinando Nicoletti**

Italy

**Marta Nieto**

Spain

**Tomoyuki Nishizaki**

Japan

**Wendy Noble**

United Kingdom

**Alexi Nott**

United States of America

**Young J. Oh**

Republic of Korea

**Seog Bae Oh**

Republic of Korea

**Masuo Ohno**

United States of America

**Toshihisa Ohtsuka**

Japan

**Hideyuki Okano**

Japan

**Takaaki Ozawa**

Japan

**Hyungju Park**

Republic of Korea

**H.R. Parri**

United Kingdom

**Stephane Peineau**

United Kingdom

**Jan Plock**

Switzerland

**Andrew Poulos**

United States of America

**Shuang Qiu**

China

**Yong Rao**

Canada

**Kimberlei Richardson**

United States of America

**Cl Roulston**

Australia

**Michael Rowan**

Ireland

**Hoon Ryu**

United States of America

**Katherine Sadleir**

United States of America

**Masanori Sakaguchi**

Japan

**Carlo Sala**

Italy

**Anupama Sathyamurthy**

United States of America

**Mako Sato**

Japan

**Shailendra Saxena**

India

**Je Hoon Seo**

Republic of Korea

**Wongi Seol**

Republic of Korea

**Chengyong Shen**

China

**Sanbing Shen**

Ireland

**Hirotaka Shoji**

Japan

**Bai Chuang Shyu**

Taiwan, Republic of China

**Jong-Woo Sohn**

Republic of Korea

**Juan Song**

United States of America

**Tinna Stevnsner**

Denmark

**Volko Straub**

United Kingdom

**Yongchao Su**

United States of America

**Byung-Chang Suh**

Republic of Korea

**Cheuk-Kwan Sun**

Taiwan, Republic of China

**Dandan Sun**

United States of America

**Chao Tai**

United States of America

**Keizo Takao**

Japan

**Kenkichi Takase**

Japan

**Tomohiko Takei**

Japan

**Xiangshi Tan**

China

**Zhi-Yong Tan**

United States of America

**Zhibin Tan**

United States of America

**Koichi Tanaka**

Japan

**Yanmei Tao**

China

**Ahmad Tariq**

United States of America

**Ayumu Tashiro**

Singapore

**Kathryn Todd**

Canada

**Hiroshi Tokumitsu**

Japan

**Ikuo Tooyama**

Japan

**Hiroki Toyoda**

Japan

**Richa Tripathi**

United Kingdom

**Hiroshi Tsuda**

Canada

**Makoto Tsuda**

Japan

**Shusaku Uchida**

Japan

**Yoichi Ueta**

United States of America

**Juzoh Umemori**

Finland

**Atsuhiko Uno**

Japan

**Luis M. Valor**

Spain

**Manlio Vinciguerra**

United Kingdom

**Noah Walton**

United States of America

**Chao Wang**

United States of America

**Tzu-Hao Wang**

Taiwan, Republic of China

**Ying Wang**

United States of America

**Xiaoming Wang**

United States of America

**Yong-Ting Wang**

China

**Yuan Wang**

China

**Ayako Watabe**

Japan

**Sumiko Watanabe**

Japan

**Zhexing Wen**

United States of America

**Albert Wong**

Canada

**Long-Jun Wu**

United States of America

**Zhigang Xiong**

United States of America

**Junyu Xu**

China

**Koji Yamanaka**

Japan

**Jun Yan**

Canada

**Riqiang Yan**

United States of America

**Yuchio Yanagawa**

Japan

**Zhengang Yang**

China

**Mark Yeoman**

United Kingdom

**Seung-Yong Yoon**

Republic of Korea

**Zili You**

China

**Seong-Woon Yu**

Republic of Korea

**Xiang Yu**

China

**Gerald W Zamponi**

Canada

**Scott Zeitlin**

United States of America

**Xiaohui Zhang**

China

**Ming-Gao Zhao**

China

**Chunjie Zhao**

China

**Yu Zhou**

China

**Feng-Quan Zhou**

United States of America

**Jiawei Zhou**

China

**Yang Zhou**

United States of America

**Min Zhuo**

Canada

